# Tracing the spread and phylogeography of foot-and-mouth disease virus across East and the Horn of Africa

**DOI:** 10.1093/ve/veaf073

**Published:** 2025-09-19

**Authors:** Dennis N Makau, Jonathan Arzt, Kimberly VanderWaal

**Affiliations:** Department of Biomedical and Diagnostic Sciences, College of Veterinary Medicine, University of Tennessee, Knoxville, Knoxville, TN 37996, United States; Department of Public Health Pharmacology and Toxicology, Faculty of Veterinary Sciences, University of Nairobi, P. O. Box 29053 - 00625 Kangemi, Kenya; Department of Veterinary Population Medicine, College of Veterinary Medicine, University of Minnesota, United States St. Paul, MN 55108, United States; Foreign Animal Disease Research Unit, U.S. Department of Agriculture-Agricultural Research Service, Plum Island Animal Disease Center, P.O. Box 848, Greenport NY 11944, USA; Foreign Animal Disease Research Unit, U.S. Department of Agriculture-Agricultural Research Service, National Bio-and Agro Defense Facility, Manhattan, 1980 Denison Ave., KS 66502, USA; Department of Veterinary Population Medicine, College of Veterinary Medicine, University of Minnesota, United States St. Paul, MN 55108, United States

**Keywords:** East Africa, Horn of Africa, foot-and-mouth disease, phylogeography, viral dispersal, genomic surveillance

## Abstract

Foot-and-mouth disease (FMD), a highly contagious viral infection affecting cloven-hoofed animals, has significant implications for global livestock production and trade. In this study, we aimed to characterize and describe dispersal patterns and factors affecting pool 4 serotypes of FMD viruses (FMDVs) in the East and Horn of Africa. The study area included 12 countries, i.e. Sudan, South Sudan, Eritrea, Djibouti, Ethiopia, Somalia (Horn of Africa) and Kenya, Uganda, Tanzania, Rwanda, Burundi, and Malawi (East Africa); 1423 VP1 sequence data were used (224 serotype A, 593 serotype O, 310 SAT1, and 296 SAT2), obtained from the National Center for Biotechnology Information (NCBI) GenBank database. Using continuous and discrete space phylogeographic models in BEAST, we assessed viral dispersal, population dynamics, direction, and velocity modelled against environmental, human, and livestock demographic and trade data as raster files. We observed a rise in accessible sequences in the last decade, signifying enhanced surveillance and research endeavours but emphasizing the need for rigorous analyses to address biases, ensuring comprehensive data collection for precise phylogeographic inference, and highlighting the importance of genomic surveillance given the geographical imbalance pre-1970. Higher precipitation correlated with increased dispersal velocity for certain serotypes, while elevation influenced the direction of viral spread. Proximity to human and livestock populations, i.e. urbanization and agricultural activities, also influenced spatial transmission dynamics. We identified distinct viral clusters with Kenya and Sudan as major sources for intercountry spread in the East and Northern regions, respectively. Regional collaboration, data sharing, and targeted surveillance, informed by genomic data and environmental factors, can aid in early outbreak detection and management.

## Introduction

Foot-and-mouth disease (FMD) is arguably one of the most economically devastating transboundary viral diseases affecting cloven-hoofed animals globally. It is caused by the FMD virus (FMDV), a member of the *Piconaviridae* family (Zell et al. 2017). FMD not only inflicts substantial losses upon livestock industries but also poses a significant threat to global trade, food security, and rural livelihoods, especially in low- and middle-income countries (Knight-Jones et al. 2017). Broadly, FMDV is classified into seven distinct serotypes (O, A, C, Asia1, SAT1, SAT2, and SAT3) (Grubman and Baxt 2004, Brito et al. 2017, Ranaweera et al. 2019). Previous studies on genetic diversity revealed distinct clades associated within different geographic regions, resulting in FMDVs found in the East and Horn of Africa as belonging primarily to pool 4, which includes Serotypes A, O, C, SAT1, SAT2, and SAT3. Serotype C is considered extinct after last being reported in 2004 in Kenya (Grubman and Baxt 2004, Sangula et al. 2011).

Over the years, pool 4 viruses have shown a propensity for sustained cocirculation and sporadic outbreaks in the region despite numerous and varying control measures instituted, including vaccination and restriction of animal movements (Hammond et al. 2021; Kerfua et al. 2018; Maree et al. 2014; Vosloo et al. 2002; Woldemariyam et al. 2023). Particularly in recent years, with the emergence of historically African strains in Asia and Türkiye, such as strains belonging to serotypes O (EA-3) and SAT2 (Knowles et al. 2005, Canini et al. 2022, Aslam and Alkheraije 2023, Duyum and Francom 2023), one can argue that the East and Horn of Africa regions are a focal point for the spread and persistence of emergent FMDV strains. Like other FMD viruses, pool 4 viruses exhibit a distinctive genetic (and antigenic) signature, which necessitates a comprehensive investigation into their phylogeography and dynamics in the region.

The East and Horn of Africa regions are characterized by complex ecological and socio-economic factors, varied livestock management practices, frequent wildlife–livestock interactions, porous intercountry borders, and varied disease management policies between countries, which all may contribute to the spread of FMDV in the region. Understanding the phylogeography and evolutionary dynamics of circulating FMDV is crucial for devising effective control strategies, including vaccination and movement restrictions in the region. To achieve this, characterizing the genetic and geographic distribution of pool 4 FMDV and identifying key factors driving these dynamics are paramount.

Several recent studies have documented some of the trends and dynamics of specific serotypes of FMDV in some countries in this region in the past; [Sudan (Lycett et al. 2019; Raouf et al. 2022), Ethiopia (Sulayeman et al. 2018, Gizaw et al. 2020, Tesfaye et al. 2020), Eritrea (Tekleghiorghis et al. 2017, Lycett et al. 2019), Djibouti (Duchatel et al. 2019, Woldemariyam et al. 2023), Somalia (Duchatel et al. 2019), Kenya (Munsey et al. 2021; Omondi et al. 2019; Omondi et al. 2020), Uganda (Velazquez-Salinas et al. 2020, Munsey et al. 2021, 2022), Tanzania (Kerfua et al. 2018, Velazquez-Salinas et al. 2020, Munsey et al. 2021), Rwanda (Duchatel et al. 2019, Udahemuka et al. 2022), and Malawi (Duchatel et al. 2019; Omondi et al. 2019)]. However, except for Duchatel et al. (2019) and Lycett et al. (2019), most studies either focused on one or two of the pool 4 FMDV serotypes in up to three countries, thus describing a partial picture of the cocirculation dynamics of pool 4 viruses in the region. In this study, we integrate molecular epidemiology with geospatial modelling to explore the factors shaping the distribution, transmission pathways, and evolution of pool 4 FMDV across 12 countries in the region, providing a more comprehensive understanding of its current spread and evolution. Through this study, we hope to disentangle the intricate interplay between host distribution, viral genetics, and geographical/environmental factors (Duchatel et al. 2019, Munsey et al. 2021, 2022), ultimately contributing to the development of more targeted and effective strategies for controlling the spread of FMD and hopefully providing insights for viral emergence.

Briefly, sequence data and relevant factors that may be associated with the spatial dispersal of a pathogen can be used in Bayesian phylodynamic models to quantitatively test the hypotheses about viral evolution and transmission, reconstruct the geographic patterns of spread, and map the flows of population (Lemey et al. 2010, 2014, Drummond et al. 2012, Alkhamis et al. 2016, Rambaut et al. 2018, Bouckaert et al. 2019, Makau et al. 2021). This analytical framework has enabled the inclusion of spatial and environmental data in phylodynamic analysis to understand the spread of different pathogens, e.g., Ebola, influenza, and HIV in humans (Dudas et al. 2017, Müller et al. 2018, Rasmussen et al. 2018); FMD and porcine reproductive and respiratory syndrome virus (PRRSV) in livestock (Duchatel et al. 2019, Makau et al. 2021, Munsey et al. 2021); and infectious wildlife diseases (Fountain-Jones et al. 2017, Yang et al. 2019). An extension to this analytical modelling framework is the addition of tools that allow for the extraction of certain analytical outputs (‘seraphim’; Dellicour et al. 2016) for further modelling the rate and direction of viral spread and the role of environmental and landscape factors.

We aimed to investigate the phylogeography and dynamics of Foot-and-mouth disease virus (FMDV) spread in the East and Horn of Africa. To achieve this, we characterized the co-circulation patterns of all pool 4 FMDV serotypes. Our study specifically aimed to analyse the temporal and spatial patterns of FMDV spread both within and between countries in the region. Additionally, we sought to identify and compare the environmental factors influencing the spatial dynamics and spread of different FMDV serotypes within the East and Horn of Africa.

## Materials and methods

### Study design and data management

**Figure 1:**
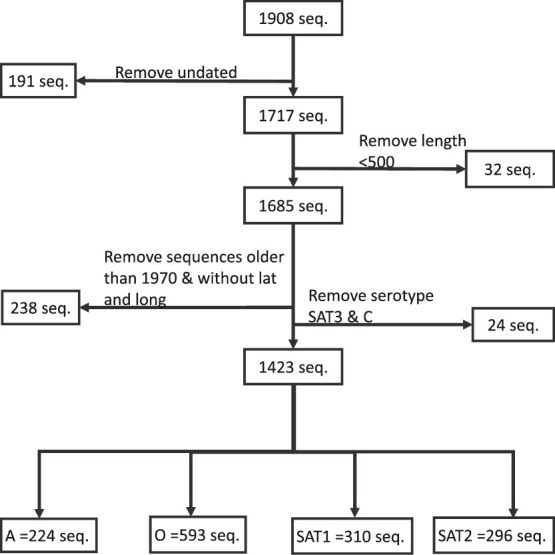
Flow diagram of data cleaning for 1908 pool 4 foot-and-mouth disease virus VP1 sequences from the East and Horn of Africa (up to 2019), resulting in a final dataset of 1423 sequences across serotypes A, O, SAT1, and SAT2.

To understand the dynamics of FMDV in this region, we considered 12 countries, i.e. Sudan, South Sudan, Eritrea, Djibouti, Ethiopia, Somalia (Horn of Africa) and Kenya, Uganda, Tanzania, Rwanda, Burundi, and Malawi (East Africa). The 12 countries were selected based on their inclusion in FMDV Pool 4, a World Organisation for Animal Health (WOAH)/Food and Agriculture Organization (FAO)-defined epidemiological zone in East Africa and the Horn of Africa. This region is characterized by endemic circulation of serotypes O, A, SAT1, and SAT2; high livestock mobility; and shared surveillance frameworks, making it a cohesive unit for studying FMDV dynamics (Wubshet et al. 2019, Woldemariyam et al. 2023). In September 2022, we retrieved 1908 VP1 sequences from the study region as a subset of 3958 annotated nucleotide sequences downloaded from the NCBI GenBank database. To maximize inclusivity given the known inconsistencies in NCBI GenBank’s metadata for geographic origin, the initial download targeted all African countries by iteratively using combinations of country names and the keyword ‘FMDV VP1’ in the GenBank search interface. No filters for serotype, host, or collection date were applied at this stage (Supplementary Table 1 and Supplementary File S1). The 1908 sequences corresponding to the Pool 4 countries were subsequently curated using the metadata, as outlined in Fig. 1. For this analysis, sequences from all host species were included, but although pool 4 viruses include serotypes A, O, C, SAT1, SAT2, and SAT3, only four of these six serotypes were included in the analysis. Serotype C was considered extinct (Sangula et al. 2011), and SAT3 sequences were too few (*N* = 3) to be included in subsequent analysis. Serotype exclusion by type was based on the corresponding metadata downloaded from the NCBI GenBank database.

Ultimately, our analysis included 1423 VP1 sequences across four serotypes: A (*n* = 224), O (*n* = 593), SAT1 (*n* = 310), and SAT2 (*n* = 296). Metadata for all sequences used and excluded are available in Supplementary Table 1 and Supplementary File S1 (Makau 2025).

**Figure 2:**
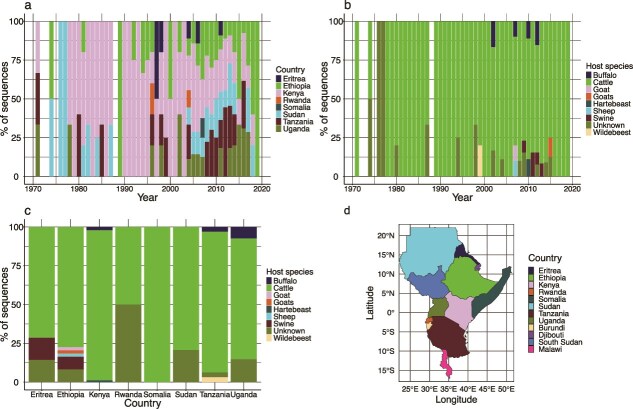
Bar plots depicting (a) proportional distribution of pool 4 FMDV sequences by country and year, (b) proportional distribution of pool 4 FMDV sequences by species and year, (c) proportional distribution of pool 4 FMDV sequences by species and country in the East and Horn of Africa between 1970 and 2019 *(N = 1423),* and (d) map of the east and horn of Africa showing total number of VP1 sequences per country (shaded), 1970–2019.

Due to the genetic and antigenic uniqueness between the serotypes, all analyses and models were serotype specific. To mitigate potential biased inference of the location of ancestral nodes during model fitting, which can result from over-representation of some locations, particularly in the earlier years of our study period, we censored samples with sampling dates prior to 1970. Post-1970, the study region saw a relatively balanced distribution of samples, with at least three countries represented in the first 5 years (Fig. 2a). While this distribution may not fully reflect the actual disease incidence across all countries, the inclusion of samples from multiple countries helped mitigate the disproportionate influence of early sequences in our analysis. This balanced representation was deemed acceptable to effectively address our research question regarding the transmission dynamics of pool 4 FMDV serotypes and associated factors. However, we acknowledge that although the application of random walk (Brownian motion) models in continuous space phylogeography is considered more robust to sampling imbalance, it does not completely eliminate biases, especially in endemic settings (Kalkauskas et al. 2021, Chen et al. 2023) such as this one, and this limitation has been acknowledged in Section 4 of this paper.

### Phylogeographic analysis

To assess the presence of a temporal signal in the phylogenetic data, we applied TempEst to serotype-specific maximum likelihood phylogenetic trees constructed using RaxML (v8) on the CIPRES Science Gateway (v 3.2) (Miller et al. 2010). These trees were derived using the general time-reversible, gamma distribution (GTR + Γ) substitution model (Rambaut et al. 2016, Munsey et al. 2021) with 100 bootstraps (Makau et al. 2021). Root-to-tip regression analyses revealed a moderate to strong temporal signal across the serotypes with positive correlations (*R* = 0.75 for serotype O; 0.86 for serotype A; 0.31 for serotype SAT1; 0.42 for serotype SAT2) (Supplementary Fig. 1). These results indicate a moderate to strong temporal signal for serotypes O and A and a weaker yet present signal for SAT1 and SAT2. Despite the lower correlation values for SAT1 and SAT2, we retained these datasets for further analysis due to their biological importance and overall data integrity. Where applicable, the influence of potential outliers was reviewed, but no sequences were excluded to artificially improve model fit. To reconstruct the spatial spread of FMDV lineages, we applied both continuous and discrete space phylogeographic methods implemented in BEAST (v 1.10.4) (Lemey et al. 2010, Suchard et al. 2018).

In the phylogeographic models, we incorporated the GTR + Γ substitution model, relaxed molecular clock model, and Skygrid coalescent population model [Bayesian Skygrid (Sg)] (Gill et al. 2013). All BEAST XML configuration files used in this study are publicly available in Zenodo 10.5281/zenodo.15659465.

#### Continuous space model

In the continuous space model, phylogenetic branches and nodes were mapped to geographic coordinates, facilitating the estimation of dispersal velocity based on the geographical distance and temporal duration of each branch (Duchatel et al. 2019, Munsey et al. 2021). In this model, we used a Cauchy relaxed random walk model for inference of the spatial locations of internal nodes (Dellicour et al. 2021), allowing for nuanced assessment of the factors influencing spatial dynamics. From this model, the dispersal velocity of a branch could be estimated from the distance between the geographical coordinates of an internal node and its descendant in the tree and the length/temporal duration of the branch. Variable dispersal velocities between different branches on a phylodynamic tree were used to evaluate the influence of environmental, climatic, and anthropogenic drivers on the speed of spatial diffusion across the landscape (Dellicour et al. 2016; Dellicour et al. 2020a).

We used the mean of the log marginal likelihood of two Markov chain Monte Carlo runs of 800 million generations, sampling every 80 000 generations run in BEAST (v1.10.4) (Suchard et al. 2018). Model convergence was assessed using Tracer (v1.7.1) by examining the effective sample size for each parameter, with values exceeding 200 indicating adequate convergence. Ten percent of the initial chain was discarded as burn-in (Drummond and Bouckaert 2015). We used the *‘seraphim’* package (Dellicour et al. 2016) in R (R Core Team 2022) for detailed spatiotemporal analysis. We randomly subsampled 1000 BEAST trees from which the posterior distribution and spatiotemporal information were extracted after ignoring 10% burn-in. In this analysis, we estimated the mean and weighted branch dispersal velocities (weighted across the tree), direction of spread, and historical patterns of spread. Spatial and environmental variables (Table 1; Pybus et al. 2012, Munsey et al. 2021) were rigorously integrated to assess their impact on the dispersal dynamics (speed and directionality) of FMDV across the landscape. The factors included: cattle, sheep, goat, and pig density (Gilbert et al. 2018); annual precipitation, precipitation seasonality, and elevation (Fick and Hijmans 2017), which may be indicators of variation in fodder availability, as well as livestock management practices and movement in pastoralist herds; distance to hubs of high human population density (generated in this study); and distance to the nearest livestock market (generated in this study). Our hypotheses around these factors, as well as data sources for the spatial raster files, are summarized in Table 1 below.

**Table 1:** Environmental factors included as covariates in phylogeographic models of foot-and-mouth disease virus (FMDV) spread in the east and horn of Africa, with data sources and hypothesized rationale

Factor raster files	Data source	Hypothesis/rationale
Livestock density Cattle Sheep Goats Pigs	Obtained from the Harvard Dataverse, V1 based on the Global cattle distribution in 2015 https://dataverse.harvard.edu/dataset.xhtml?persistentId=doi:10.7910/DVN/LHBICE https://dataverse.harvard.edu/dataset.xhtml?persistentId=doi:10.7910/DVN/VZOYHM https://dataverse.harvard.edu/dataset.xhtml?persistentId=doi:10.7910/DVN/CIVCPB https://dataverse.harvard.edu/dataset.xhtml?persistentId=doi:10.7910/DVN/YYG6ET	We predicted that the host density in a given area would influence dynamics of spread whereby higher dispersal velocities may be inferred in areas with high cattle, sheep, goat, and pig densities, as these represent FMDV-susceptible livestock kept in the region and involved in transboundary movements.
Climatic data Annual Precipitation Precipitation seasonality	Obtained from the list of bioclimatic variables in the WorldClim 2 database at a 1 km spatial resolution climate surfaces for global land areas. bioclim code BIO12 https://www.worldclim.org/data/worldclim21.html bioclim code BIO15 https://www.worldclim.org/data/worldclim21.html	Differences in precipitation may influence dynamics of viral spread as areas with higher rainfall/more stable precipitation may have less animal movement, which will impact viral epidemiology as herds contact and disperse to and away from communal grazing and watering areas. Moreover, seasonality may impact animal movements and between-herd contacts in pastoralist settings, which could influence the speed and direction of viral spread (Muleme et al. 2012, Hamoonga et al. 2014, Munsey et al. 2019, Munsey et al. 2022).
Elevation	Obtained from the list of bioclimatic variables in the WorldClim 2 database at 1 km-spatial-resolution climate surfaces for global land areas. https://www.worldclim.org/data/worldclim21.html	High-elevation areas may experience higher precipitation and are characterized by smaller land holdings often managed using intensive production systems. This affects animal movements, potentially influencing the spread of diseases, much like precipitation and seasonality patterns. Therefore, we hypothesize that elevation may positively or negatively influence the rate of viral spread.
Distance to human population hubs with: ≥1% of country population; ≥2% of country population; ≥5% of country population	Hub distance calculated in QGIS based on data obtained from known towns/cities from Google and country-level population from worldometer (https://www.worldometers.info/world-population/) Supplementary Fig. 2	In the region, urban hubs with up to 1% of the country’s population were major towns and cities, while hubs with 2% were country capitals and large commercial centres, and hubs with 5% of the population were exclusively capitals in 8 of the 12 countries. Due to incompleteness of animal movement records in the region, we hypothesize that animal movements would be influenced by supply chains and demand for meat in urban centres and towns. Potentially, the direction and spread of viral spread may be correlated with the distance to these human population hubs and markets (Muleme et al. 2012, Munsey et al. 2021, 2022).
Distance to main livestock markets	Location of livestock markets in the regions obtained by getting coordinates from Google Maps search using the term [**main livestock markets in ‘paste country name’**] and hub distance calculated in QGIS Supplementary Fig. 3	A study in Uganda observed that viruses stay closer to livestock markets than spread outwards from the markets. Since livestock markets are points of livestock aggregation, we hypothesized that some of animal movements would be influenced by trade and since not all markets were located in population hubs, a separate distance metric specific to markets would provide some additional insights. Potentially, the direction and spread of the virus may be correlated with the distance to and from markets (Muleme et al. 2012, Motta et al. 2019, Munsey et al. 2021, 2022).

##### Dispersal directionality of FMDV serotypes

We applied functions included in the *‘seraphim’* package to assess whether there were differences between trends of ‘preferential/resource-driven’ spread of FMDV serotypes in the study region. The suite of functions in *‘seraphim’* allows for the testing of whether the presence, absence, or variation of certain attributes of a geographical location results in either viruses remaining in areas with similar attributes or moving away from them (Dellicour et al. 2019). The difference between this analysis and the dispersal analysis in 2.5 is that the branch durations, path, and transition times are not considered; instead, the metrics are calculated with reference to the geographical origin and destination of the nodes in the phylogenetic tree. In this analysis, two metrics were calculated, the mean environmental values (*E*) calculated at each node position measuring the tendency of lineages (nodes) to remain in locations with higher/lower values, and *R*, the proportion of branches for which environmental values at the ancestral nodes was higher/lower than at descendant nodes (approximating the tendency to disperse towards locations with higher/lower values) (Munsey et al. 2021). Subsequently, a distribution was generated after computing these values for each BEAST tree sampled and compared to the null distribution (randomizing the phylogenetic node positions within the study area, assuming constant branch lengths, tree topology, and root position). Thus, we assessed the potential role of each spatial factor to draw viruses to or away from a given location.

##### Dispersal velocity of FMDV serotypes

To ascertain whether specific factors influenced the rate of viral spatial diffusion within the region, we assessed how distinct factors enhance or diminish the speed with which FMDV disperses in the region. In this analysis, each branch on the time-scaled phylogenetic tree was considered a movement vector with location coordinates (latitude and longitude) defining the start and end points for specific nodes (viruses) through time. As such, with branch-specific distance and time, we were able to calculate the velocity of viral dispersal in the landscape. These movement vectors were then assigned an environmental path distance between start and end points of the branch weighted on the basis of the underlying landscape attributes from an embedded raster of specific factors described in Table 1. The changing values along the branch may affect how easily the virus moves across the landscape from its starting point to its destination. To compute these values, we used the least-cost path model (Dijkstra 1959) and the Circuitscape path model (McRae 2006).

The least-cost path model applies an algorithm to determine the least-cost route (i.e. the route that minimizes the summed values of raster cells traversed between locations), whereas the Circuitscape path model uses circuit theory to account for uncertainty in the path taken. We evaluated all factors both as conductors (spatial factors associated with increasing velocity of viral spread) and as resistors (reducing velocity of viral spread). We calculated *Q*, the difference between a regression coefficient obtained from branch durations against environmental distances computed on the raster being evaluated as a predictor vs. obtained by regressing branch durations against a null raster in which all raster values were set to 1 (i.e. duration is solely the function of distance traversed). Ultimately, a Bayes factor (BF) was obtained by calculating the statistical support of each positive *Q* distribution evaluated against a null distribution generated by a randomization procedure in which the phylogenetic node positions within the study area are randomized, keeping branch lengths (time and distance), tree topology, and root position constant (Dellicour et al. 2016, 2017, Munsey et al. 2021).

#### Discrete-space analysis

For this analysis, we employed a discrete space phylogeographic model, wherein each branch of the phylogenetic tree represents a temporal span and the likely discrete location—specifically, the country—is inferred for each internal tree node. This approach allowed us to map the transboundary spread of the virus over time (Duchatel et al. 2019, Munsey et al. 2021). Using the *‘Babel’* package in BEAST v2.5 (Bouckaert et al. 2019), we calculated the number of transitions between countries across the trees, which represent instances of transboundary viral dissemination. This calculation was performed by tracking the changes in inferred locations across the posterior distributions of BEAST-generated trees for each FMDV serotype. The transitions were quantitatively analysed to determine the frequency and patterns of viral movement between countries. We summarized the number of transition events as overall average counts and frequency distributions over time. This analysis helped in identifying the most frequent pathways of viral movement, as well as temporal trends in viral spread.

## Results

Based on the VP1 sequences obtained from the NCBI GenBank database, the representativeness of sequences across countries in the regions and the number of available sequences had increased considerably in the last 10 years (Supplementary Fig. 4 and Fig. 2a, respectively). Additionally, the representativeness of samples available for this analysis was better after 1970, prior to which available sequences were primarily only from Kenya (Supplementary Fig. 4). Moreover, there appears to be a cyclical 5-year temporal window during which most sequences are uploaded onto the NCBI GenBank database, followed by periods of very few sequences (Supplementary Fig. 5). The majority of the sequences available on the NCBI GenBank database were from cattle, with wildlife sequences primarily from African buffalo (Fig. 2b). Swine-origin sequences were exclusively sourced from countries in the Horn of Africa (Fig. 2c).

### Continuous space phylogeographic inference

**Figure 3:**
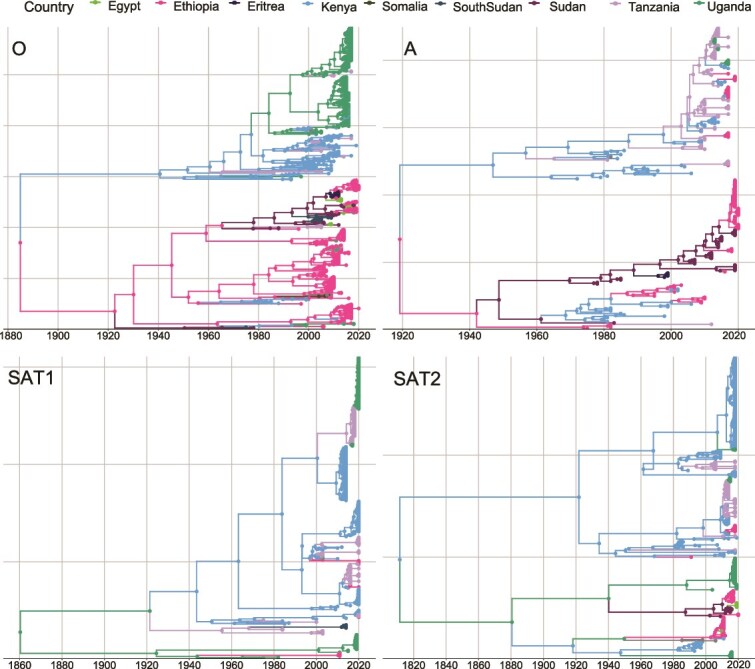
Country-annotated time-scaled phylogenetic trees for foot-and-mouth disease virus (FMDV) pool 4 serotypes circulating in the East and Horn of Africa up to 2019.

From the continuous space beast models, it was evident that the most common recent ancestor (time to the most recent common ancestor, tMRCA) for FMDV in the region for the different serotypes was the late 1800s for three of four serotypes. The tMRCA of serotype O was 1883 (95% HPD 1810–1928) with an almost immediate differentiation into two major clades associated with the East Africa and Horn of Africa, respectively (Fig. 3). The tMRCA for serotype A was more recent, 1918 (95% highest posterior density (HPD) 1856–1955), and also appeared to differentiate into two distinct region-specific clades, with one clade strictly circulating in Sudan and Ethiopia and the other clade in Sudan and other countries. The larger of these two clades differentiated ~20 years later into two more regionally specific clades, one involving Sudan, Eritrea, and Ethiopia (Horn region) and the other circulating between Sudan, Kenya, Uganda, and Tanzania (Fig. 3). The estimated tMRCA for SAT1 in the region was 1859 (95% HPD 1725–1945). Similar to serotypes A and O, there appears to have been an immediate differentiation into two clades, the smaller clade strictly circulating in Uganda and Ethiopia, while the other clade circulated in Kenya, Uganda, and Tanzania only (Fig. 3). The estimated tMRCA for SAT2 was 1806 (95% HPD 1616–1940). Similar to the other serotypes, at the onset, there were two distinct clades with one clade strictly circulating between Kenya, Tanzania, and Uganda (East Africa), while the other clade included Kenya, Uganda, Sudan, Rwanda, and Ethiopia (Fig. 3).

### Between-country transition inference

**Figure 4:**
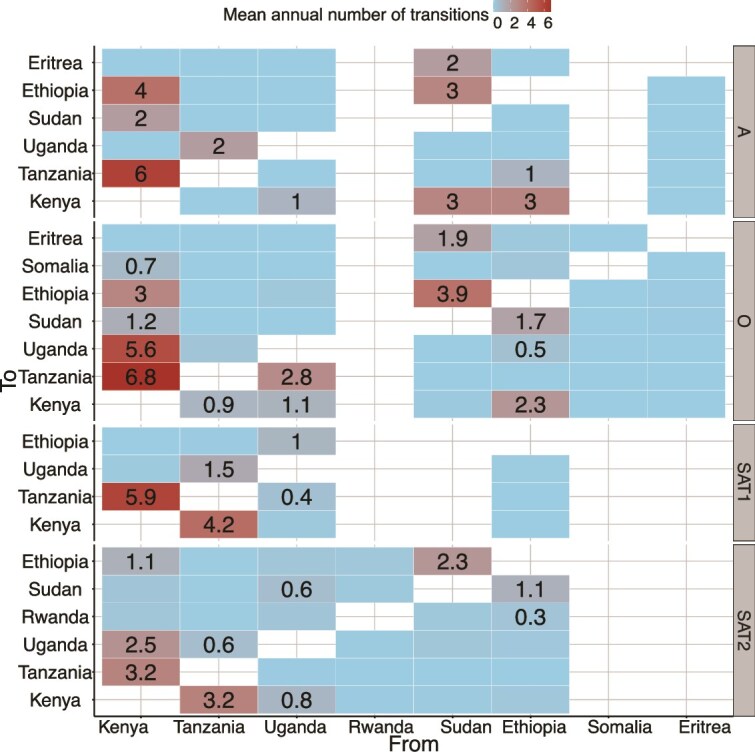
Heatmap of mean intercountry transitions of pool 4 foot-and-mouth disease virus (FMDV) across East and Horn of Africa countries, based on sequences collected between 1970 and 2019.

As expected, the majority of the dispersal occurred within specific countries, ranging between 2.1 and 421.9 transitions/year, with a mean and median of 119.3 ± 120.8 and 86.3 km/year, respectively. Of interest was how transboundary transition events varied in the region for the different serotypes. In summary, there appeared to be two broad clusters for viral dissemination, ‘eastern’, i.e. Kenya, Uganda, and Tanzania, and northern (Sudan, Ethiopia, Eritrea, and Kenya), for the sequences obtained between 1970 and 2019. More specifically, Kenya was estimated to be a major source for viruses from all four serotypes crossing to Tanzania, Uganda, and Ethiopia with mean transitions up to 7.0 annually. On the other hand, Sudan was the major origin of all (except SAT1) viruses in the ‘northern’ area (Fig. 4). Notably, Rwanda and Somalia contributed only single serotypes to the dataset used in this analysis (SAT2 and O, respectively).

### Trends of viral dispersal velocity

**Figure 5:**
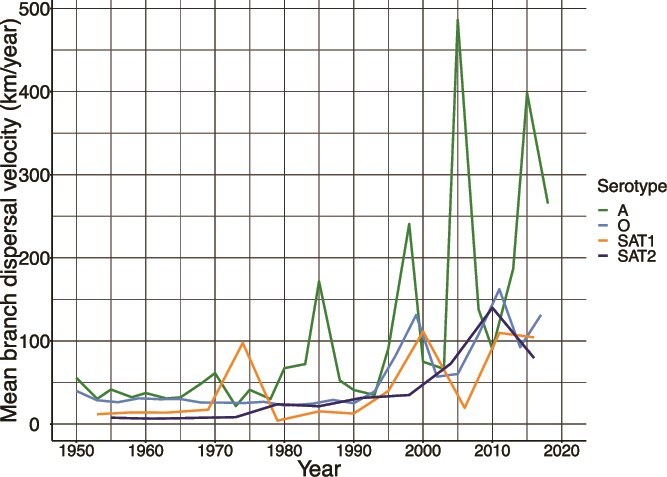
Comparative line graph of mean branch velocities for foot-and-mouth disease virus (FMDV) pool 4 serotypes in the East and Horn of Africa, with the x-axis restricted to 1950–2019 for clarity.

We observed that the mean branch dispersal velocity for all serotypes was not constant and was punctuated by periods of rapid and slow spread (peaks and dips) (Fig. 5). Of the four serotypes, serotype A had the highest mean branch velocity for the study period after 1980 (Fig. 5 and Table 2). Serotype O had the second (on average) highest mean branch velocity throughout the study period. For a more robust comparison of dispersal velocities between serotypes, we also calculated weighted branch dispersal velocities (velocity weighted by branch time) and diffusion coefficients. The dispersal velocities for serotypes A and O were almost equal, at an estimated weighted mean branch velocity of 52 and 46 km/year, for serotypes A and O, respectively (Table 2). Serotypes SAT1 and SAT2 had substantially slower dispersal velocities.

**Table 2:** Dispersal statistics for pool 4 foot-and-mouth disease virus (FMDV) serotypes in the East and Horn of Africa, estimated through continuous phylogeographic inference using sequences collected between 1970 and 2019

Serotype	A	O	SAT1	SAT2
Mean branch velocity (95% HPD) (km/year)	186.7 (183.8, 511.5)	98.2 (69.3, 218.4)	201.7 (119.2, 617.2)	95.4 (60.9, 211.9)
Mean weighted branch velocity (95% HPD) (km/year)	52.5 (39.7, 58.3)	46 (41.8, 53.5)	24.4 (17.1, 33.8)	17.6 (11.3, 26.2)
Mean diffusion coefficient (95% HPD) (km^2^/year)	22899.0 (19940.7, 70561.1)	86993.9 (47458.3, 231708.7)	34374.7 (12 429, 170 134)	9438.7 (5080.1, 28454.3)
Velocity–population size correlation (*P*-value)	0.42 (0.002)	0.39 (0.13)	0.24 (0.14)	0.39 (0.003)
Velocity–population lagged correlation—Spearman’s rho (# of years)	0.57 (9)	1.00 (5)	0.93 (6)	0.50 (6)
Velocity–distance correlation—Spearman’s rho (*P*-value)	−0.13 (<0.001)	0.33 (<0.001)	0.01 (0.39)	−0.02 (0.01)

To infer the impact of inferred viral population size and geographic extent of samples on the observed trends of dispersal, we conducted several correlation analyses. We juxtaposed annual unweighted mean branch dispersal velocities with the respective effective population sizes across years to assess the correlation between observed dispersal trends and estimated viral population in current and lagged time periods of ±5 years. Moderate positive correlations between mean dispersal velocity and estimated viral population sizes (no time lags) were observed for all serotypes except SAT1. Based on lagged correlation analysis, expansions of the effective viral population occurred 5–9 years after increases in dispersal velocity, with the strongest correlation coefficients observed for serotypes O and SAT1 (Spearman’s rho = 1 and 0.9, respectively) (Table 2). Velocity was also moderately correlated with sample size (Spearman’s rho = 0.37). Similarly, we also assessed the correlation between orthodromic geographic distance between nodes and average branch dispersal velocity. There was a weak Spearman positive correlation for serotype O (rho = 0.33), higher velocity for geographically farther sequences and a weak negative correlation for serotype A, rho = −0.13 (higher velocity for geographically closer sequences), between the orthodromic distance between tip locations and the mean dispersal velocity observed in a given year (Table 2).

### Factors affecting viral dispersal direction and velocity

As a rule of thumb, for BF interpretation, values >3 are considered ‘statistically significant’ i.e. indicative of strong support of the association between a model covariate and outcome. For these analyses, a cutoff BF value of 6.0 is recommended for more definitive interpretation of the impact of specific factors on the direction of viral dispersal. In this study, there were some notable differences between the impact of the varied factors on viral dispersal direction and location for specific serotypes.

**Figure 6:**
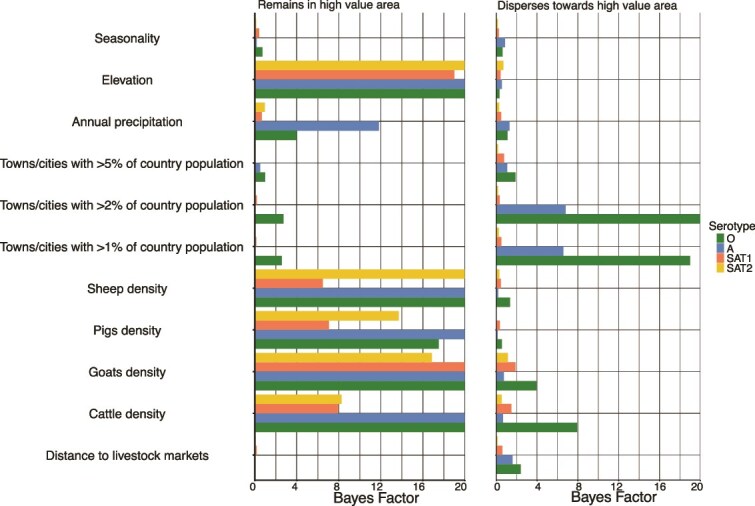
Summary statistics of impact of specific factors on dispersal direction of pool 4 foot and mouth disease viruses estimated from continuous phylogeographic inference using sequences obtained between 1970 and 2019 from the East and Horn of Africa. The left panel shows how strongly each factor supports the likelihood that livestock infected with each serotype remains in areas of high value. The right panel illustrates the support for each factor in the dispersal of livestock towards high-value areas.

Particularly, our results show the tendency of serotype O to remain in areas with higher annual precipitation (rainfall) (BF = 11.8) (Fig. 6). Also marginally supported (BF = 4.0) was the influence of annual precipitation on serotype A, also mostly circulating in areas of high precipitation. Elevation had a strong influence in the direction of viral dispersal for all serotypes, all of which tended to remain in areas of high elevation (BF > 19) (Fig. 6). Additionally, proximity to areas of human population centres was a key factor for two of the four serotypes (A and O), with viruses in these serotypes tending to disperse away from urban centres (cities with at least 1% of the country’s population) (BF > 6.0). The density of all livestock species was influential to the dispersal direction of all serotypes, with viruses tending to stay in areas of high livestock density.

**Figure 7:**
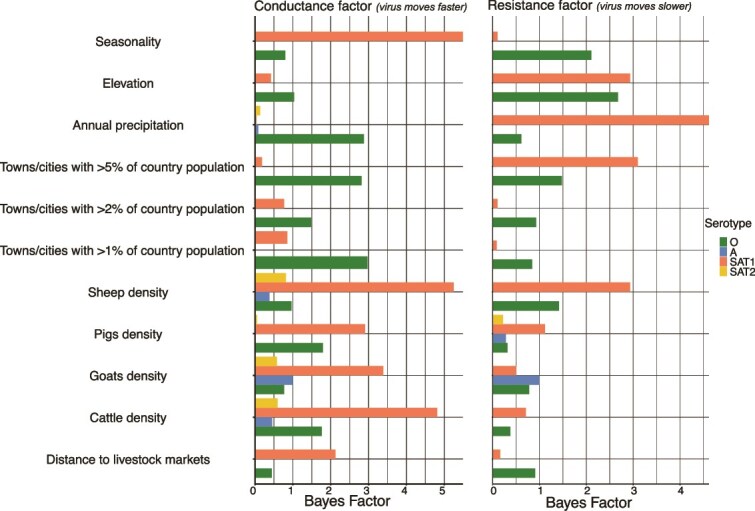
Summary statistics of impact of specific factors on the dispersal velocity of pool 4 foot-and-mouth disease viruses estimated from continuous phylogeographic inference using sequences obtained between 1970 and 2019 from the East and Horn of Africa. The left panel shows the Bayes factors indicating the strength of evidence that each listed factor facilitates faster movement of the virus across the population. The right panel similarly displays the Bayes factors for factors that are associated with slowing down the virus transmission.

Unlike the directionality analysis, few factors were identified as having an impact (BF > 3) on how fast dispersal occurred through the landscape (Fig. 7). Of note, there was marginal support that annual precipitation was associated with higher dispersal velocity for serotype A viruses (BF = 2.9) but slower dispersal of SAT1 viruses (BF = 4.6). Greater seasonality in precipitation seemed to correlate more strongly with increased dispersal velocity for SAT1, indicating a stronger association with seasonal patterns (BF = 5.5). Distance to urban hubs with at least 5% of the country’s human population seemed to slow the dispersal of SAT1 (BF = 3.1) while enhancing the dispersal of serotype A viruses (BF = 2.8) (Fig. 7). There were no BF values >3 for SAT2. Additionally, there was support that livestock densities in general enhanced dispersal (Fig. 7).

## Discussion and conclusion

### Overview

In this study, we have comprehensively characterized the complex multifactorial dynamics of FMDV spread within the East and Horn of Africa. Our findings underscore the significant influence of environmental and human factors, alongside livestock density, on the dispersion patterns of various FMDV serotypes. We have extended the existing body of knowledge by delineating how these factors concretely influence viral transmission pathways, thereby enhancing our understanding of the phylogeographic spread of FMDV (Canini et al. 2022).

We observed some level of clustering of the major viral clades in the eastern and northern countries with occasional crossovers via Kenya. High mean branch velocity (speed of diffusion) was correlated with an expected expansion of viral population 5–9 years later, which could either be an artefact of sample availability across space and time or represent true disease dynamics. From these data, the fastest-spreading serotypes in the region are serotypes A and O at a velocity of ~50 km per year based on mean weighted branch velocities (Dellicour et al. 2020b).

### Influence of environmental and climatic factors

The spread and dynamics of FMDV serotypes are significantly shaped by environmental variables such as precipitation and elevation. We observed that higher livestock densities are correlated with greater dispersal velocities, suggesting that areas with dense livestock populations are critical nodes of FMDV transmission (Fick and Hijmans 2017, Gilbert et al. 2018). Moreover, serotypes A and O exhibited a tendency to disperse towards areas with higher precipitation, which introduces a nuanced understanding of how climatic factors likely influence host behaviour and ecological conditions, indirectly shaping viral dispersal dynamics (Fick and Hijmans 2017). This could be associated with patterns of livestock movement or variation in husbandry systems. Among pastoral communities, livestock tend to move towards areas with better vegetation and water resources at certain times of the year (the same is expected for wildlife), increasing opportunities for herd mixing and interaction, as well as viral transmission and dispersal when herds move in different directions afterwards. Additionally, areas with higher precipitation in the study region are mostly characterized by intensive/semi-intensive livestock production systems with minimal livestock movement (especially cattle). While we acknowledge that the majority of sequences in this study were from cattle compared to buffalo sequences, we were unable to assess the assumed serotype host predisposition and the role of wildlife *versus* livestock spread in the direction of FMDV transitions in the region. Elevation was a strong influence on the dynamics of spread for all pool 4 serotypes. This association between higher elevation and viruses tending to remain in those areas may be related to resource availability, particularly vegetation for cattle and husbandry practices as described above.

The lower mean weighted branch velocities observed for SAT1 and SAT2 (Table 2) indicate a more localized spatial diffusion pattern compared to serotypes A and O. Although we did not explicitly stratify by wildlife versus livestock hosts, it is notable that SAT serotypes—particularly SAT2—are historically linked to long-term persistence in wildlife populations such as African buffalo (Maree et al. 2016; Vosloo et al. 1996). These wildlife hosts typically exhibit restricted ranges and limited movement, which may underlie the slower geographic spread of SAT serotypes. In contrast, the higher branch velocities for serotypes A and O are consistent with their association with extensive livestock movements and broader, transboundary epidemic dynamics. While these interpretations are limited by the quality and completeness of host metadata in our dataset, they underscore the importance of integrating host ecology into future phylogeographic studies of FMDV spread.

Proximity to urban centres was an important factor for two of the four serotypes (A and O), with viruses in these serotypes tending to disperse away from densely populated centres. While this observation suggests a potential role of human activity and urban centres in shaping viral transmission dynamics, it was counterintuitive. In our hypothesis, larger human population hubs like capital cities and commercial hubs would function as terminal points of livestock supply chains or represent increased demand for animal protein, which would result in an influx of livestock movements towards the cities/hubs for slaughter(Gerken et al. 2022, Ekwem et al. 2023). This flow of hosts would cause the virus to spread towards these urban areas (Gerken et al. 2022). However, the converse was observed from this analysis. We posit that this observed trend was associated with practices of livestock trade, where animals are mixed in live animal markets usually closer to the urban centres but those not sold from the slaughter end up moving away to respective homes postmarket days (Motta et al. 2019, Munsey et al. 2022). Regional or main livestock markets may be located in or close to major population hubs. While animals traded in these major markets could be mainly for human consumption, they could also be for further trade (Motta et al. 2019). However, the impact of these factors on direction of spread was evident.

### Transboundary spread

There are distinct regional patterns of viral transmission and spread, with Kenya and Sudan (Canini et al. 2022) serving as major hubs for specific serotypes. Our study outlines two broad clusters for viral dissemination: an ‘eastern’ cluster encompassing Kenya, Uganda, and Tanzania and a ‘northern’ cluster including Sudan, Ethiopia, Eritrea, and Kenya. Clustering of transboundary viral dissemination within geographical regions suggests that regional dynamics may be influenced by local ecological and socio-economic factors. However, given the porosity of borders and livestock movement patterns between countries (especially between immediate neighbours), intervention measures may be more effective when encompassing multiple countries in the region rather than localized at the country level. Understanding the patterns of viral spread and identifying major sources of transmission can inform targeted intervention strategies and help mitigate the impact of the disease on livestock populations and agricultural economies.

### Limitations

Research based on secondary data can be challenging. Despite targeted efforts to mitigate the impact of data limitations, the gaps in available genetic data could influence the inferred phylogenetic trees, potentially skewing the estimated tMRCAs and perceived viral spread pathways (Tatem et al. 2006, Geoghegan and Holmes 2018). The data available prior to 1970, primarily from Kenya, reflect imbalanced sampling that biases phylogeographic inference, particularly in discrete space analysis, where over-represented countries or the location of the earliest sequences may unduly influence the determination of the most likely ancestral locations (Tatem et al. 2006, Geoghegan and Holmes 2018). Our decision to censor pre-1970 data, where only Kenya was represented, may not completely overcome this sampling challenge Kalkauskas et al. 2021. For instance, Kenya’s prominent role as a source of viral transitions (Fig. 4)—particularly into Tanzania—may reflect both its central role in regional livestock trade and an overrepresentation of pre-2000 sequences in GenBank, as seen in Fig. 2a, particularly in discrete phylogeographic models. This dual influence underscores the need for more balanced molecular surveillance across neighbouring countries as surveillance biases can shape perceived epidemiological dynamics, even when real transboundary movement patterns also support such trends.

The scarcity of sequences from several regions within our study area, such as Rwanda and Somalia, which contributed sequences from only a single serotype (SAT2 and O, respectively), underscores the significant data gaps in molecular disease surveillance across the East and Horn of Africa (Kalkauskas et al. 2021, Chen et al. 2023). Further, due to reliance on the descriptions provided by original submitters on the NCBI GenBank database, the resolution of sequence localization was at times limited to county or district levels, as per author descriptions. This restriction potentially impacts the precision of our phylogeographic conclusions and might limit the interpretation of virus spread dynamics at a more localized scale. This shortfall restricts our capacity to paint a comprehensive picture of FMDV dynamics, thereby limiting our ability to accurately predict and manage future spread patterns. As part of our exploratory analyses, we also examined temporal stratification of inferred inter-country transitions in a subset of posterior trees. While some variation was observed between pre- and post-2000 periods—potentially reflecting shifts in surveillance, control efforts, or viral spread—this assessment was limited to serotype O. To avoid overinterpretation, we chose not to emphasize these preliminary findings. A more comprehensive cross-serotype comparison would be needed to robustly assess temporal trends in viral movement.

Species-level stratification observed in these data also highlights potential biases in sampling and surveillance efforts, which may influence our understanding of viral diversity and transmission dynamics. Notably, although swine production is a major agricultural practice in the East Africa region, particularly in Uganda, and ‘extensively managed’ domestic swine are known to roam freely in parts of Kenya and other parts of East Africa, swine sequences were exclusively sourced from countries in the Horn of Africa (Atherstone et al. 2019), (Thomas et al. 2013, Payne et al. 2021). This suggests notable differences in surveillance and reporting of FMD cases in different species, especially swine populations.

To effectively address these limitations, there is a critical need for enhanced surveillance and systematic genomic data collection. Strengthening collaborative networks to enhance data sharing and expanding sequencing capacities across all affected countries in the region are crucial steps forward. Establishing more comprehensive data collection protocols will also be vital for improving the robustness and applicability of our phylogeographic conclusions. Enhanced data collection efforts, including the integration of sequence-free samples from undersampled areas, will be beneficial to affirm or refute the observed viral spread and direction dynamics (Kalkauskas et al. 2021, Chen et al. 2023). These limitations notwithstanding and the analytical considerations made to mitigate obvious potential biases, we note that certain countries in the region play a crucial role in the spread of FMDV and that certain host and environmental factors differentially influence the spatial diffusion dynamics of the distinct pool 4 serotypes. However, further research with more representative data would be more informative.

## Supplementary Material

Supplementary_materials_20250611_veaf073

## Data Availability

All data used in this manuscript are publicly available in the NCBI GenBank database and websites referenced in the manuscript. Accession numbers for sequences used have been listed in the supplementary materials and are available on Zenodo 10.5281/zenodo.15659465.
