# Supplementary material for: Tracing the spread and phylogeography of foot-and-mouth disease virus across East and the Horn of Africa
Source: Virus Evol. 2025 Sep 19;11(1):veaf073. doi: 10.1093/ve/veaf073 (PMC12507016; doi:10.1093/ve/veaf073)
Supplement: Supplementary_materials_20250611_veaf073 [file supplementary_materials_20250611_veaf073.pdf]

Supplementary materials

Table 1. Example entries from the full metadata of 3,958 FMDV VP1 sequences. Columns: GenBank Accession, Strain, Serotype, Collection Date, Country, Host, Sequence Length. For full metadata (3,958 entries with accession numbers), see Supplementary File S1 or https://github.com/drdmakau/PEDIL-Phylogeography.

Strain Name	Virus Type	GenBank Accession	Sequence Length	Collection Date	Host	Country
SAT 1 Uga 1/07	Foot-and-mouth disease virus - type SAT 1	HM067706	7651	1/17/2007	Bovine	Uganda
KEN/3/57	Foot-and-mouth disease virus - type SAT 2	AJ251473	7774	-N/A-	Unknown	Kenya
SAT1-3swa1/49	Foot-and-mouth disease virus - type SAT 1	AY593840	8179	-N/A-	Unknown	Namibia
SAT1rhod5/66	Foot-and-mouth disease virus - type SAT 1	AY593846	8133	-N/A-	Unknown	Zimbabwe
SAT3-4bech 1/65	Foot-and-mouth disease virus - type SAT 3	AY593853	8131	-N/A-	Unknown	Botswana
A/Egy/2006/iso_Ism	Foot-and-mouth disease virus - type A	EF159977	802	-N/A-	Unknown	Egypt
SAT3-3kenya 11/60	Foot-and-mouth disease virus - type SAT 3	AY593852	8164	-N/A-	Unknown	Kenya
UGA/09/2004	Foot-and-mouth disease virus - type SAT 2	GU323177	347	2004	Unknown	Uganda
SAR/19/2000	Foot-and-mouth disease virus - type O	AJ539140	8184	-N/A-	Unknown	South Africa
A21 Kenya	Foot-and-mouth disease virus - type A	AY593761	8161	-N/A-	Unknown	Kenya
UGA/03/2004	Foot-and-mouth disease virus - type SAT 2	GU323173	347	2004	Unknown	Uganda
SAT 2 Uga 2/07	Foot-and-mouth disease virus - type SAT 2	HM067704	7642	1/17/2007	Bovine	Uganda
UGA/05/2004	Foot-and-mouth disease virus - type SAT 2	GU323174	344	2004	Unknown	Uganda

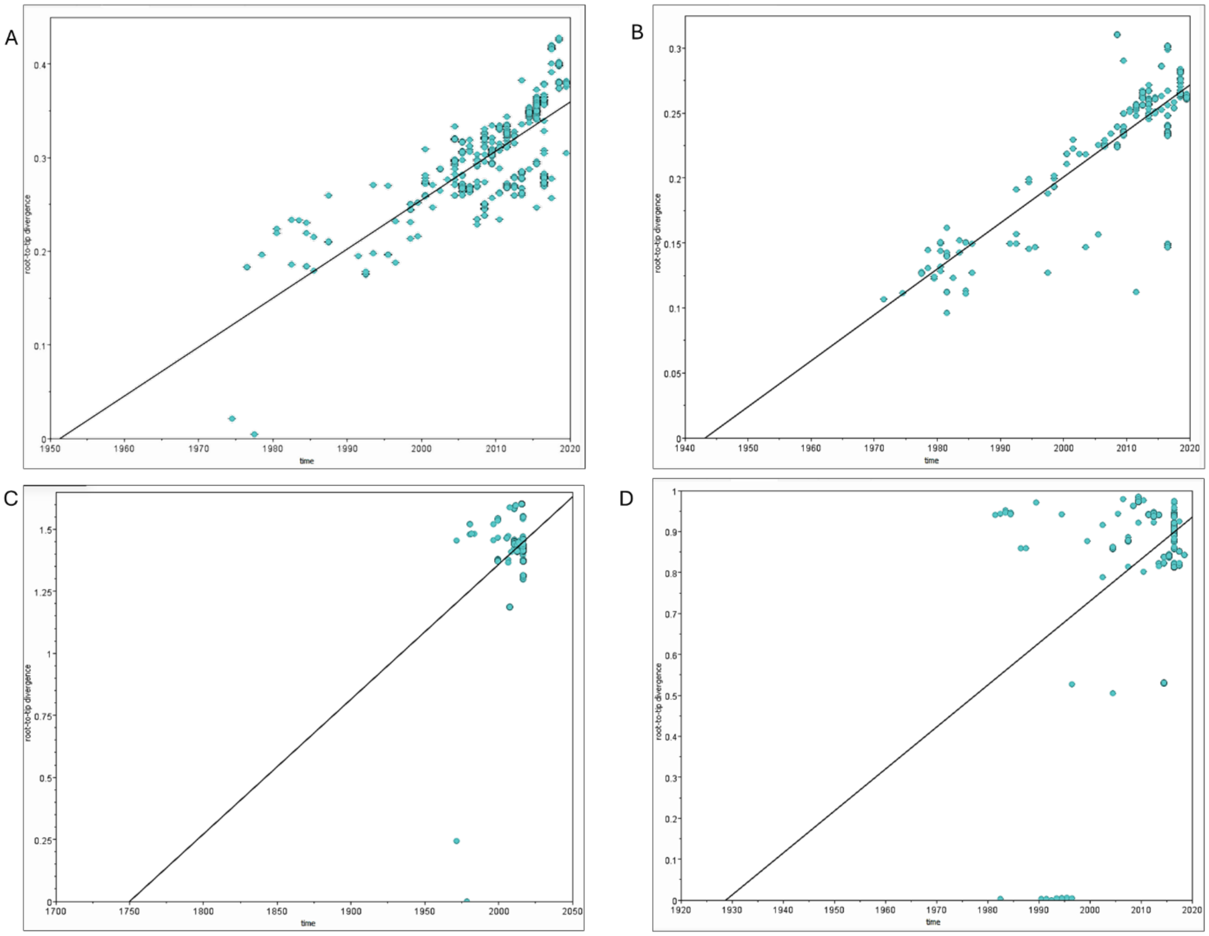

Supplementary figure 1. Root-to-tip regression plots for each FMDV serotype showing temporal signal in the sequence data. Maximum likelihood phylogenetic trees were inferred using RAxML under a GTR+Γ model and analyzed in TempEst. Each plot displays the linear relationship between genetic divergence (root-to-tip distance) and sampling time for (A) serotype O [N=593], (B) serotype A [N=224], (C) serotype SAT1 [N=310], and (D) serotype SAT2 [N=296]. The strength of the temporal signal is indicated by the correlation coefficient (R) and coefficient of determination (R^2). Serotypes O and A exhibited strong temporal structure (R = 0.75 and 0.86, respectively), while SAT1 and SAT2 showed weaker correlations (R = 0.31 and 0.42, respectively). These results support the use of molecular clock models for downstream time-scaled phylogeographic analyses.

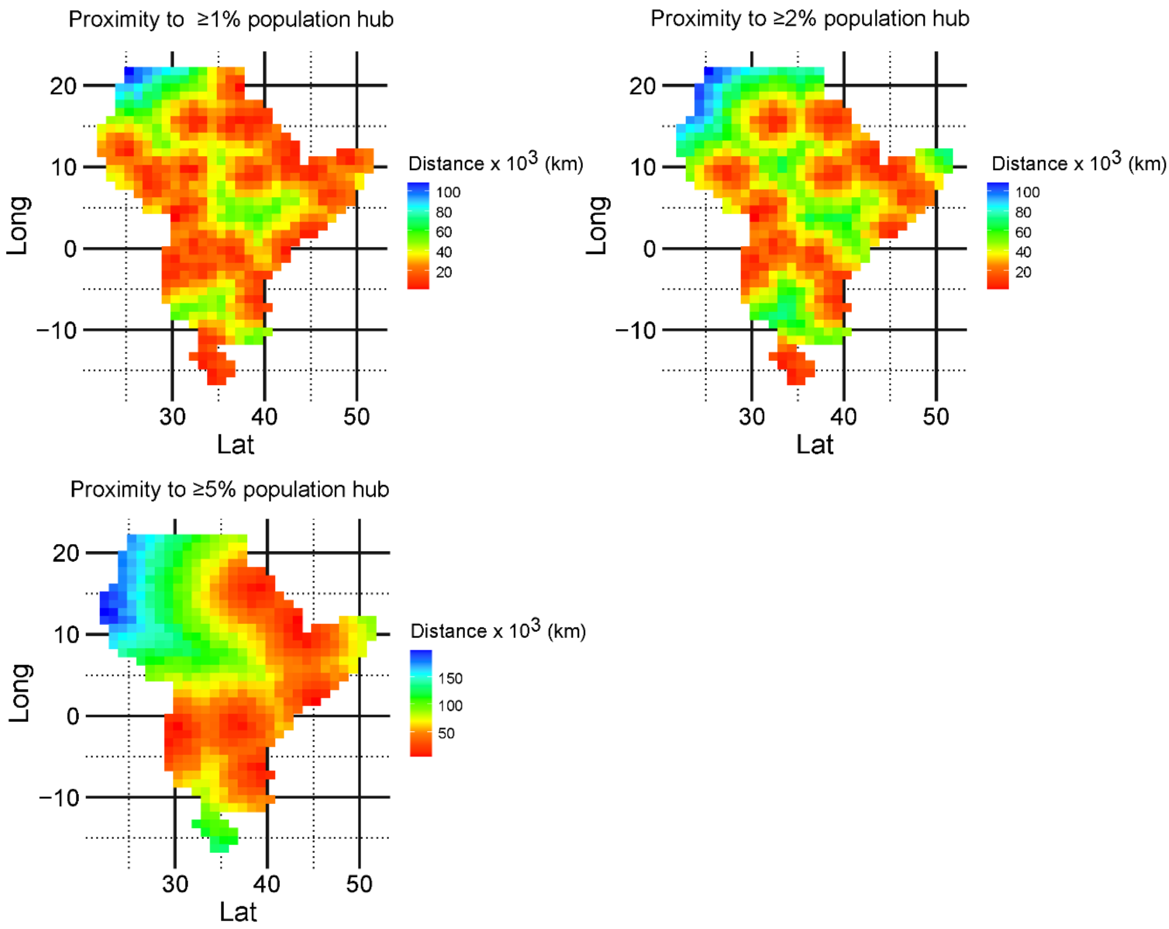

Supplementary figure 2: Map depicting hub distance (proximity to) population hubs with up to 1-5% of country population in East and Horn of Africa. Hotter colors representing closer shorter distances

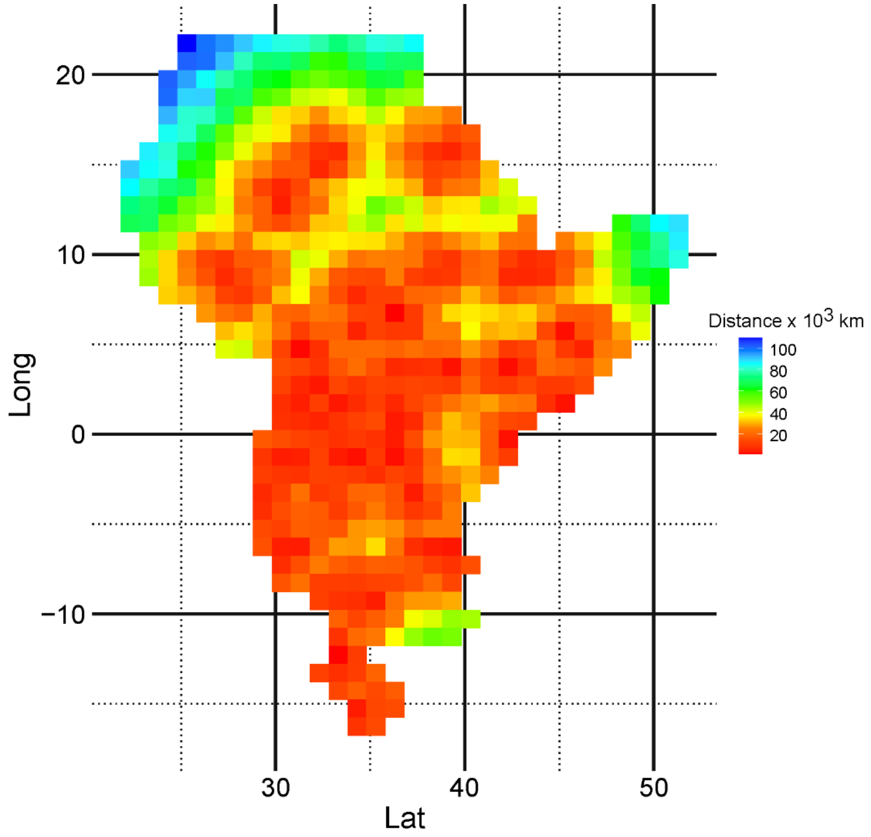

Supplementary figure 3: Map depicting hub distance (proximity to) to major livestock markets in East and Horn of Africa. Hotter colors representing closer shorter distances

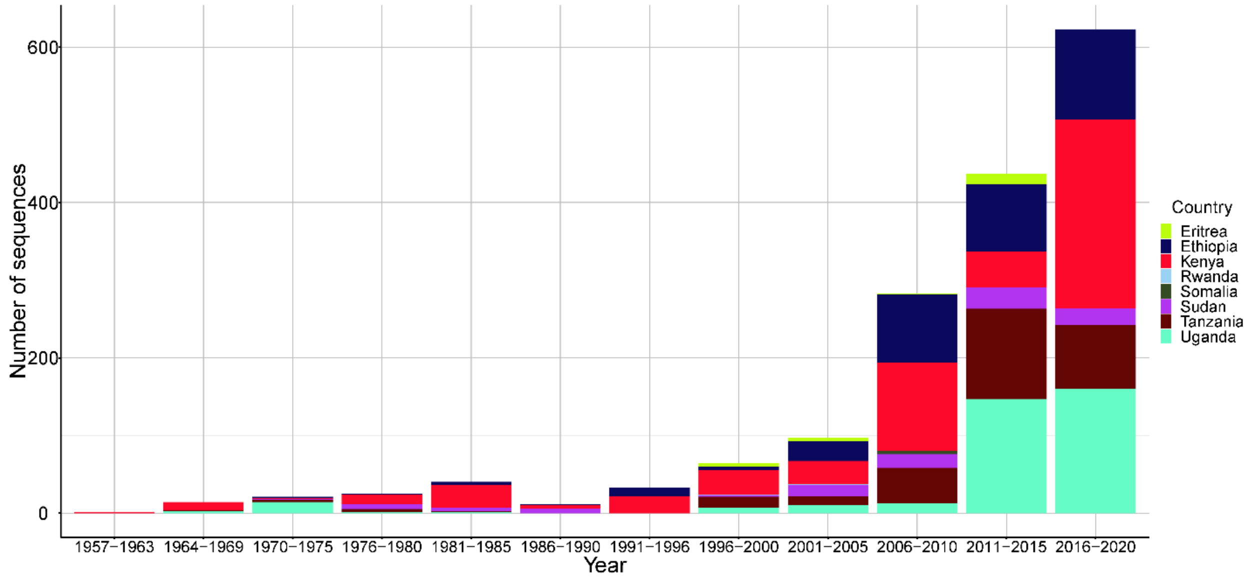

Supplementary figure 4: Distribution of VP1 FMDV sequences in the East-and-Horn of Africa region downloaded from GenBank East and Horn of Africa between 1970 and 2019 *(N= 1685).*

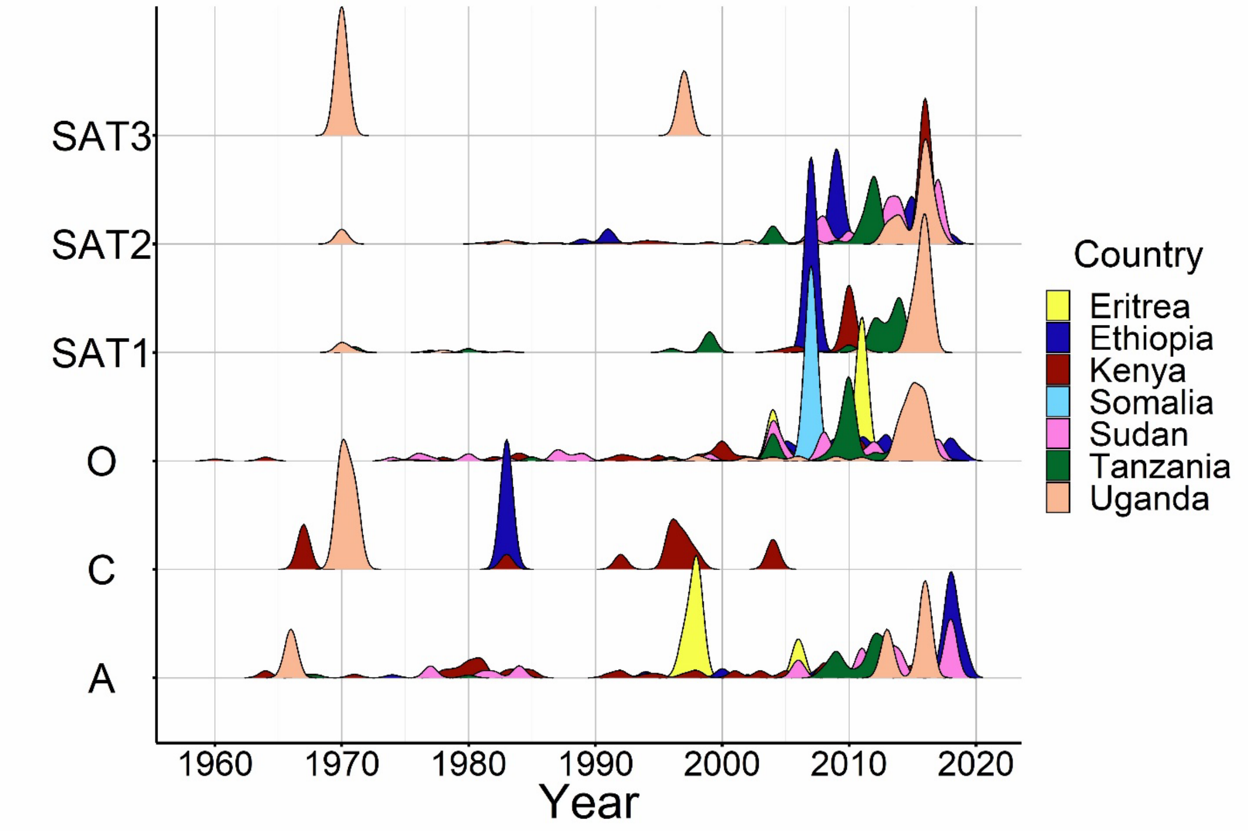

Supplementary figure 5: Joyplot (ridgeline plot) showing the temporal distribution of VP1 sequences of Foot-and-Mouth Disease Virus (FMDV) by serotype and country across the East and Horn of Africa. The data represent sequences downloaded from GenBank spanning 1970 to 2019 (N = 1,685). Curve heights reflect relative sampling density and are not scaled to absolute counts; the plot is intended for visual comparison of temporal and geographic sampling coverage rather than quantitative interpretation.
